# 

**Published:** 1867-10

**Authors:** 


### OHIO COLLEGE OF DENTAL SURGERY.

THE regular annual session of this Institution will commence on the 15th inst., and continue five months, one month longer than heretofore; this extension having been made by the association of the Colleges of Dentistry.

The curriculum of this Institution has undergone a very material change since its last session; this occurs in part by the action of the above named association, and in part by the action of the Ohio Dental College Association, together with that of the Board of Trustees and the Faculty.

In making these changes, the only object was to secure a more thorough course of instruction, to more thoroughly prepare the student for usefulness and efficiency in the practice of his chosen profession.

By a division of the course into two classes, less labor is given to the student than heretofore, which was acknowledged to be overtaxing and unreasonable. Now, with fewer branches, and longer time, far more thorough acquaintance with these branches will be a necessary result; and besides, the same ground will not be traveled over twice.

There are now eight teachers, instead of five or six as formerly.

All the fundamental branches of medicine are thoroughly taught. Anatomy, Physiology, Histology, Pathology, Therapeutics, Inorganic and Organic Chemistry, will each be presented in from sixty to seventy lectures. These branches are taught not only as to the general physician, but in addition to this, their application to the practice of the Dental specialty.

Every Dentist thoroughly skilled in the science of his profession will recognize the fact at once, that there are many men thoroughly familiar with all these branches, and yet quite unfit for Dental practitioners. The truth of this statement is most fully evidenced by the mistakes made, and the errors maintained in regard to the teeth, their diseases and treatment, by some of our profoundest medical men. And hence the necessity for a special teaching, for the application of a knowledge of these things to the practice of this specialty.

### NOTICE.

The Transactions of the last meeting of the American Dental Association, held in Cincinnati, from July 30th, to August 3d, inclusive, are now ready for distribution, and will be sent immediately to the members entitled to them; they are also for sale to all others who may desire to purchase them, at $3.00 per volume.

### TENNESSEE DENTAL ASSOCIATION.

THIS Society was organized at Nashville on the 26th day of July, 1867, with a very good representation of the profession of the State of Tennessee present. A Constitution, By-laws, and Order of Business were framed and adopted; the whole of which we regard as very good. The regular meetings are to be held semi-annually in December and July; at such places as may be designated by the Association. Dr. W. H. Morgan, of Nashville, is President, and with him is associated a splendid corps of officers.

Thus the organization of Associations goes on, and from it an incalculable amount of good will result. Every State should, and we believe soon will, have its Dental Association, in which all the legitimate part of the profession ought to be embraced. In that way, and that only, can the profession in the different sections become harmonious in feelings, principles, and practice.

### The Dental Register

OF THE WEST.

*Issued Monthly, at $3 a Year in Advance.*

DEVOTED TO THE INTERESTS OF THE DENTAL PROFESSION.

EDITED BY J. TAFT AND G. WATT.

The volume commences in January and closes in December.

The Register being issued monthly is always fresh, therefore indispensable to the practitioner who desires to keep posted up with the new inventions and improvements which are daily developed. Neither expense nor effort will be spared to give value and variety to its contents. Articles will be illustrated with well executed engravings whenever they will add to the interest or assist in explanation.

Its extensive circulation and frequency of issue makes it one of the BEST DENTAL ADVERTISING MEDIUMS in the United States.

RATES OF ADVERTISING.

One page, 1 year, $50. Half page, 1 year, $30. Quarter page, or card, 1 year $20.

All advertising bills payable in advance, or at furthest after the issue of the first number containing the advertisement. All transient advertisements to be paid for in advance.

☞ CONTRIBUTIONS SOLICITED.

Address, J. TAFT, or "DENTAL REGISTER," Cincinnati Ohio.

Business Communications to

J. TAFT, Publisher,
No. 238 Fourth St., between Plum and Central Avenue

